# Supplementary material for: Causal interplay between lactose intolerance and gut microbiota: a combined bidirectional Mendelian randomization and in vivo validation study
Source: Front Nutr. 2026 Jun 1;13:1803337. doi: 10.3389/fnut.2026.1803337 (PMC13265576; doi:10.3389/fnut.2026.1803337)
Supplement: Supplementary file 1 [file Data_Sheet_1.zip › supplementary materials/Forward/leave one out cross validation/ebi-a-GCST90027719.finngen_R12_E4_LACTONAS.pdf]

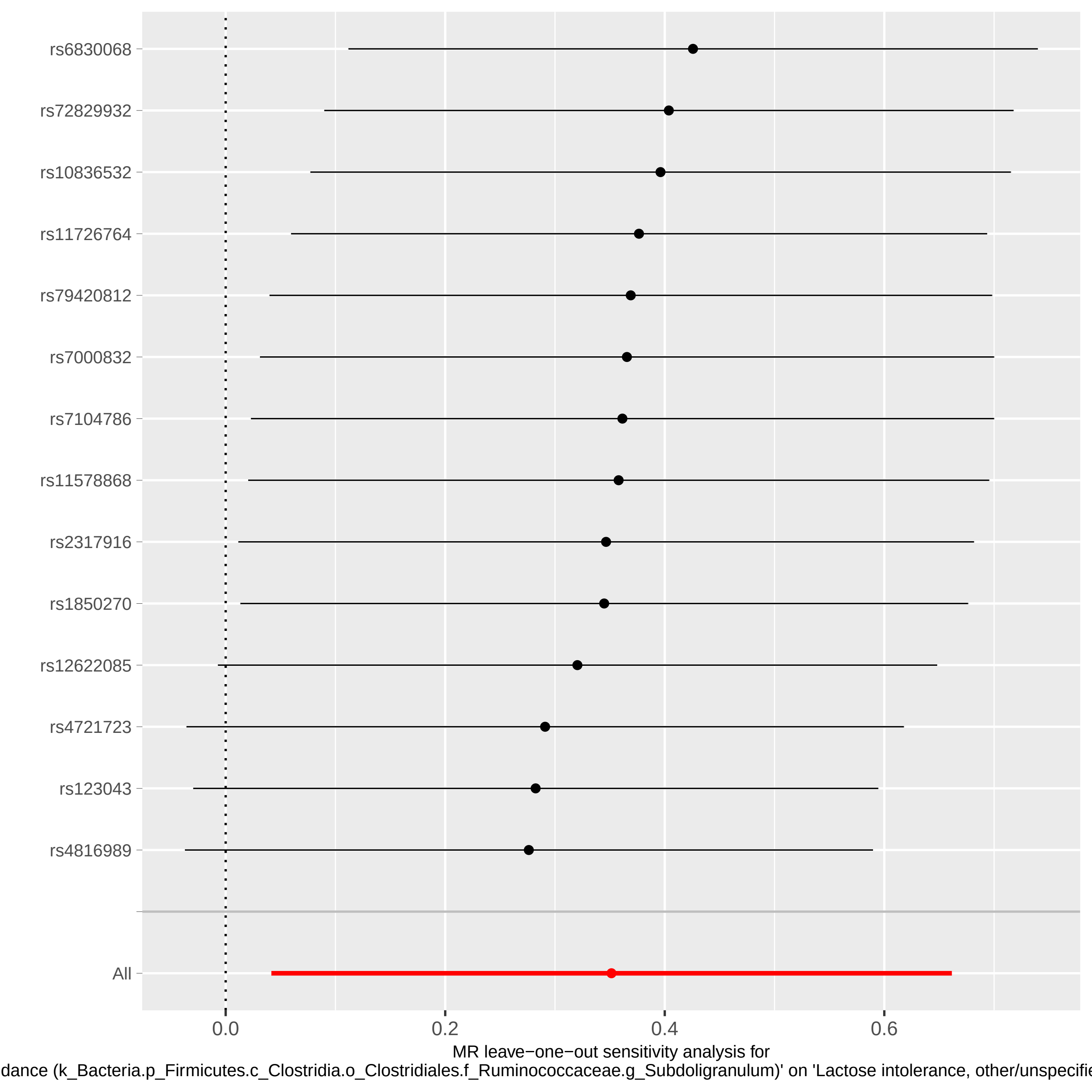

rs6830068
rs72829932
rs10836532
rs11726764
rs79420812
rs7000832
rs7104786
rs11578868
rs2317916
rs1850270
rs12622085
rs4721723
rs123043
rs4816989
All
0.0
0.2
0.4
0.6
MR leave−one−out sensitivity analysis for
dance (k_Bacteria.p_Firmicutes.c_Clostridia.o_Clostridiales.f_Ruminococcaceae.g_Subdoligranulum)' on 'Lactose intolerance, other/unspecifie
